# Morphometric Identification, Gross and Histopathological Lesions of *Eimeria* Species in Japanese Quails (*Coturnix coturnix japonica*) in Zaria, Nigeria

**DOI:** 10.1155/2014/451945

**Published:** 2014-11-05

**Authors:** H. A. Umar, I. A. Lawal, O. O. Okubanjo, A. M. Wakawa

**Affiliations:** ^1^Department of Veterinary Parasitology and Entomology, Faculty of Veterinary Medicine, Ahmadu Bello University, Zaria 2222, Nigeria; ^2^Department of Avian Medicine, Faculty of Veterinary Medicine, Ahmadu Bello University, Zaria 2222, Nigeria

## Abstract

The objective of the study was to identify the species, gross and histopathological lesions of *Eimeria* in Japanese quails in Zaria. A total of 400 fresh faecal samples were collected and 10 quail birds were purchased from a quail farm. The faecal samples were processed using simple floatation technique. Oocysts shape indices of sporulated oocysts were determined. The intestines were observed for gross lesions and segments were analyzed using Giemsa stain and Haematoxylin and Eosin stain and then observed microscopically for the developmental stages of the parasite. Four species of *Eimeria* were identified in the study. *Eimeria bateri* of shape index of 1.36 conformed to the guidelines used while the other three *Eimeria* species with shape indices of 1.48, 1.03, and 1.40 were not confirmed. The main gross lesion seen was nonhaemorrhagic ballooning of the caeca. Intestinal scrapping smear revealed a developmental stage of the parasite (merozoites) in the jejunum. Histopathology also revealed a developmental stage (schizont) of the parasite in the caecum and desquamation of the epithelial lining with areas of necrosis. Further study is required using molecular techniques to properly identify the unknown species of *Eimeria* that were detected in the study.

## 1. Introduction

Quails are most susceptible to various diseases such as coccidiosis which is recognized as a serious parasitic disease problem limiting quail industry [1]. Quail production has become important in Nigeria. Descriptions of* Eimeria* date from the beginning of the last century, and ever since means for an appropriate characterization and identification of the species have been discussed. Several parameters can be used [2] and new methods have been developed [3–6]. Various species of* Eimeria *have been isolated from the different species of quails such as* E*.* tsunodai*,* E. uzura,* and* E. bateri *described from Japanese quails [7] and* E. lophortygis *and* E. okanaganensis *described from California quails, while* E. crusti *and* E. oreortygis *are described from mountain quail [8],* E. conturnicis *and* E. bateri *are described from grey quail [9],* E. colini *and* E. lettyae *are described from bob white quail [10], and also* E. tahamensis *is described from Arabian quail [11]. The natural infection of* Eimeria* in quails is characterized as subclinical because of the mild and nonspecific clinical signs. Nevertheless, coccidiosis is considered as an important disease because the endogenous stages of the parasites and a high number of oocysts in feces are associated with intestinal lesions [7]. Therefore, the objective of the study was to identify the species and report the gross and histopathological lesions of* Eimeria *in Japanese quails in Zaria, Nigeria.

## 2. Materials and Methods

### 2.1. Study Area

This study was carried out in Zaria located in Kaduna State, located within latitudes 11°7′ to 11°12′ N and longitude 07°41′ E. It is a medium sized city with an estimated population of 408,198 [12]. It is divided administratively into Zaria and Sabon Gari LGAs [13]. It has an estimated land area of about 300 square kilometers and it is approximated that about 40–75% of its working population derive their principal means of livelihood from agriculture [14]. Zaria which is located in the North Guinea Savannah zone of Nigeria has an annual ambient temperature, ranging between 18.0 ± 3.7°C and 31.8 ± 3.2°C. The harmattan season (the cold-dry period of the year) lasts from November to February, while the hot-dry season lasts from March to May and the rainy season lasts from June to October [15, 16].

### 2.2. Sample Collection, Handling, and Processing

Forty fresh faecal samples were collected using polythene bags. Ten live quail birds were also gotten from the farm. The samples were labeled and transported to the Helminthology Laboratory in the Department of Veterinary Parasitology and Entomology, Faculty of Veterinary Medicine, Ahmadu Bello University, Zaria. In the laboratory samples not examined immediately were refrigerated at 4°C to maintain the integrity of the oocyst [17].

### 2.3. Laboratory Examination

The faecal samples were examined for the presence of coccidia oocysts using the simple floatation technique as described by Urquhart et al. [18].

Oocyst positive samples were diluted into 2.5% aqueous potassium dichromate (K_2_Cr_2_O_7_) and kept in Petri dishes for sporulation at room temperature. After sporulation, oocysts were recovered by centrifugation with saturated sugar solution as described by Duszynski and Wilber [17] and used in subsequent analysis.

The shape indices (length/width) of the sporulated oocysts were determined by using the method for species identification as described by Harper and Penzorn [19].

The calculated oocysts shape index values were then compared with the standard diagnostic guide provided by Teixeira et al. [20] to determine the species encountered in the study.

### 2.4. Necropsy

The birds were euthanized and this was accomplished in accordance with guide for the care and use of laboratory animals [21].

Segments of the small intestine (duodenum, jejunum, and ileum) and large intestine (caeca) were taken for gross and histopathological studies.

The segments were examined macroscopically for gross lesions. Intestinal scraping smear was carried out to detect the presence of developmental stages of the parasite within the intestine using Giemsa stain as described by Dubey [22].

Histological examination was carried out to confirm the presence of the developmental stages of the parasites and pathological lesions within the small and large intestine using Haematoxylin and Eosin stain as described by Mitchell et al. [23].

### 2.5. Statistical Analysis

Oocysts measurements were analyzed using the software Excel (Microsoft) for mean and standard deviation.

## 3. Results

### 3.1. Oocyst Speciation

Table 1 shows the oocyst dimensions and morphological characteristics of different* Eimeria* species identified in the study. The* Eimeria* species encountered in the study were* Eimeria bateri *(Figure 2) and three other* Eimeria *species (Figures 3, 4, and 5) not covered by the diagnostic guidelines.

### 3.2. Gross and Histopathological Lesions

**Figure 1:**
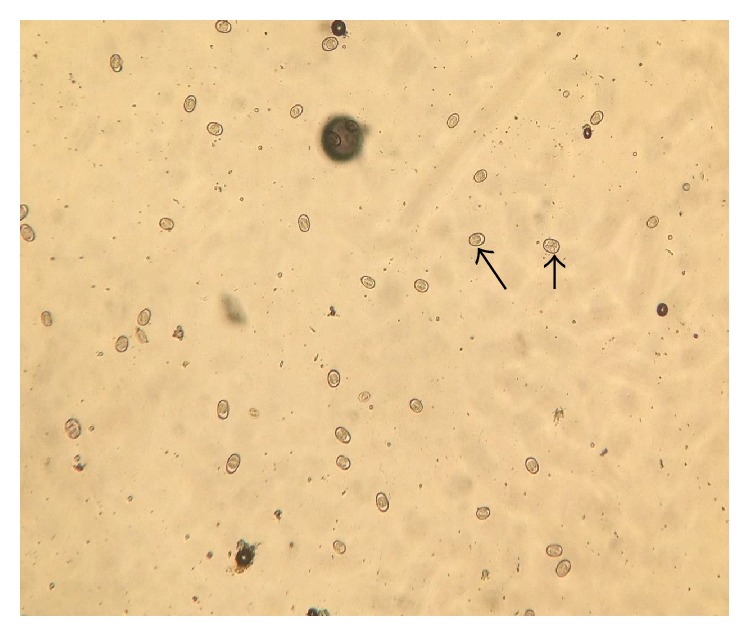
Numerous unsporulated* Eimeria* oocyst from quails using simple floatation technique (×10).

**Figure 2:**
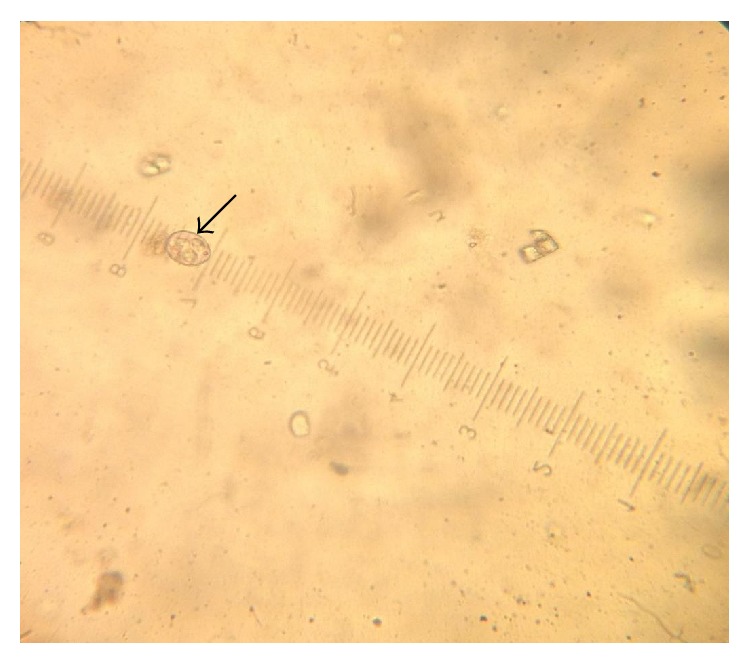
Subspherical* Eimeria bateri* oocyst with a polar granule using ocular micrometer (×40).

**Figure 3:**
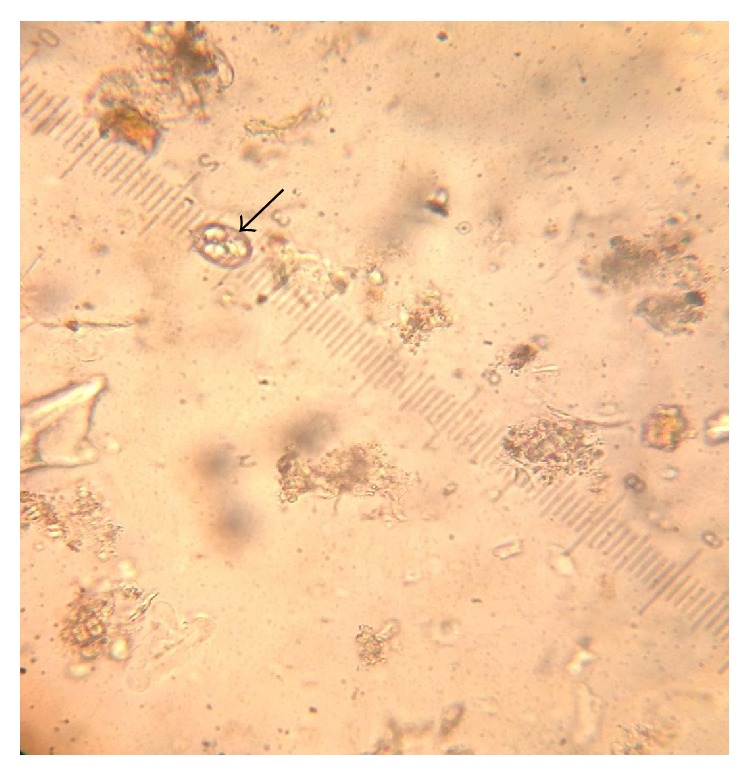
Ovoid* Eimeria* oocyst using ocular micrometer (×40).

**Figure 4:**
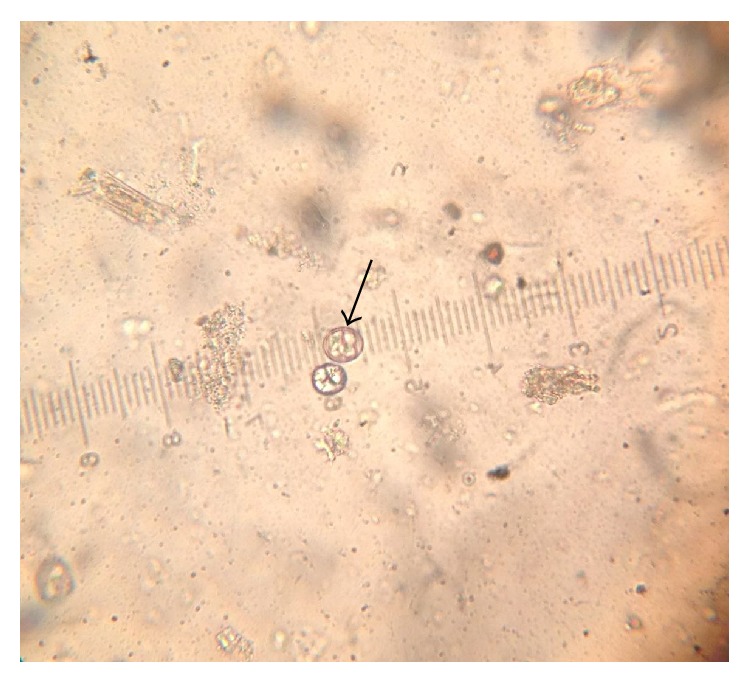
Ellipsoidal* Eimeria* oocyst using an ocular micrometer (×40).

**Figure 5:**
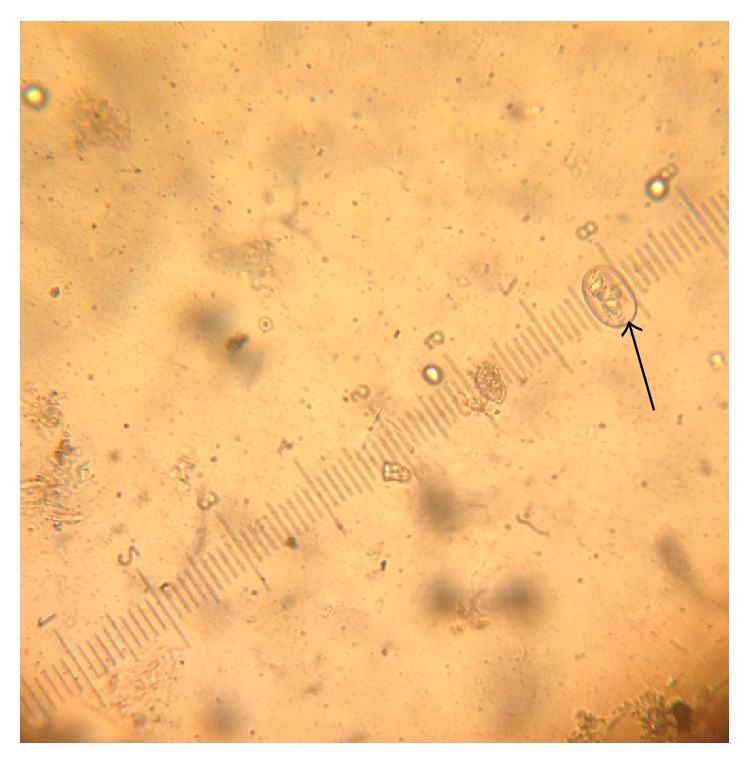
Subspherical* Eimeria *oocyst with no polar granule using ocular micrometer (×40).

**Figure 6:**
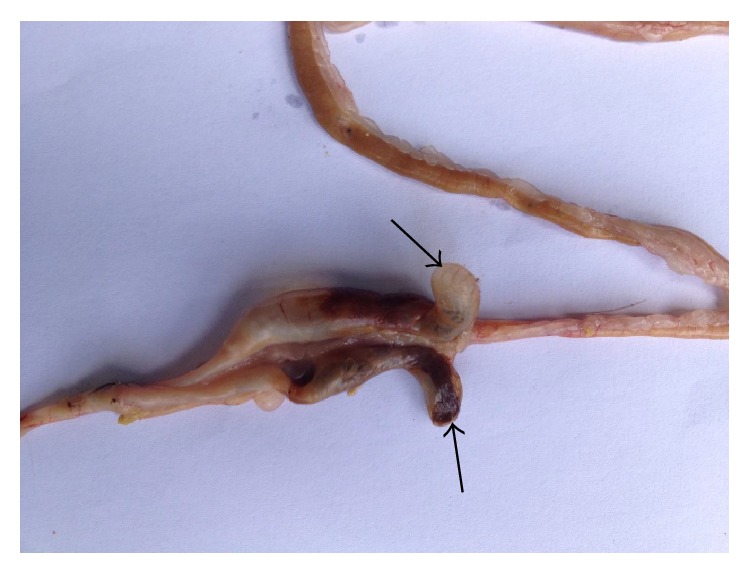
Ballooning of the caeca of a Japanese quail naturally infected with* Eimeria bateri.*

**Figure 7:**
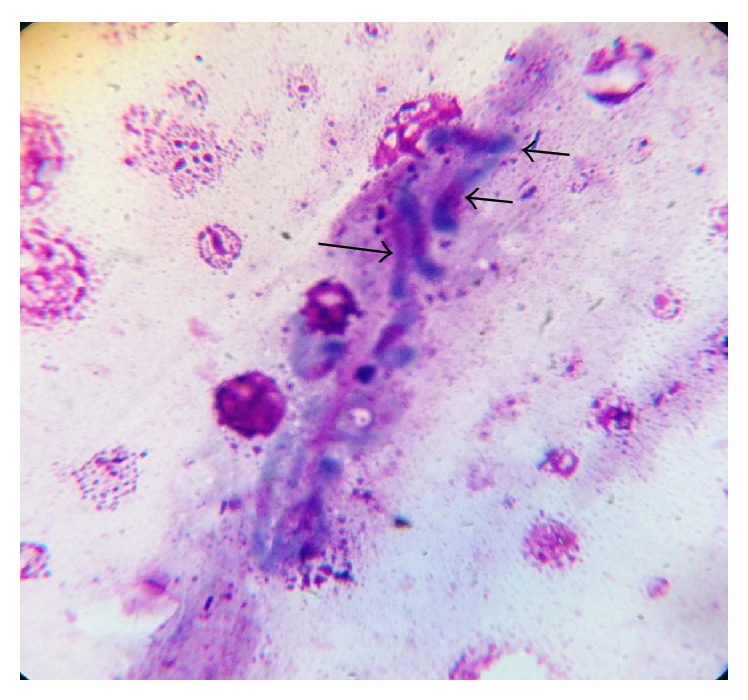
Jejunum of Japanese quail showing the developmental stage (merozoite) of* Eimeria *spp. in the epithelium using Giemsa stain (×100) oil immersion.

**Figure 8:**
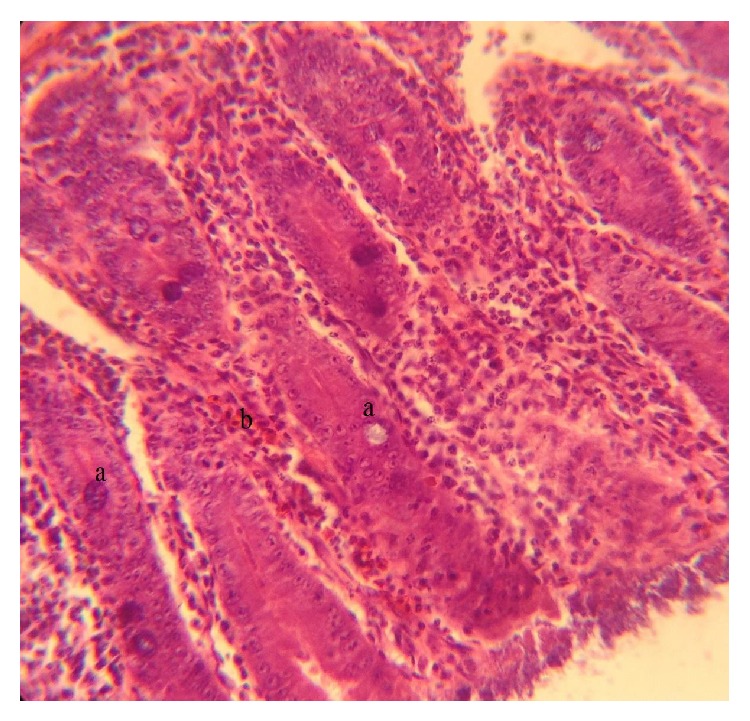
Histological section of the caecum showing the (a) developmental stage (schizont) of* Eimeria *spp. and (b) area of necrosis H&E (×40).

**Figure 9:**
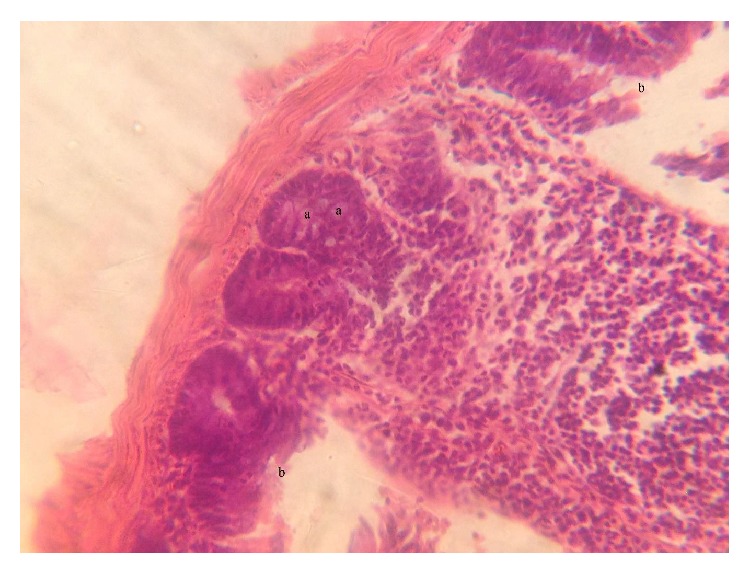
Histological section of duodenum showing the (a) developmental stage (schizonts) of* Eimeria *spp. and (b) desquamation of the epithelium H&E (×40).

The fresh faecal sample was positive for* Eimeria *oocysts (Figure 1). The main gross pathology seen was the ballooning of the caeca which contains no bloody exudate in the lumen (Figure 6). Histopathologically, desquamation of intestinal mucosa was observed (Figure 9) and caecal necrosis (Figure 8). Developmental stages of the parasite especially the merozoites and schizont were seen in the intestinal epithelium (Figures 7, 8, and 9).

## 4. Discussion

Quails are considered a branch of the modern poultry industry in Nigeria. The study showed that there were coccidia oocysts in the farm sampled from. In our study the identification of* Eimeria* species was done using the oocysts morphometric technique. Four* Eimeria *species were identified from Japanese quails in the study.* Eimeria bateri* with shape index of 1.36 conformed to the guidelines of Teixeira et al. [20] and Mohammad [24]. The other three* Eimeria* species with shape indices of 1.48, 1.03, and 1.40 could not speciate as they do not conform to any of the given species by the above authors.

In spite of the shortcomings identified above, differences in morphology of the oocyst which were ovoid, subspherical, and ellipsoidal, presence and absence of polar granule can be observed. This means that other unidentified species of* Eimeria* exist in Zaria. Discrimination of* Eimeria* species using this technique has sort of limitations to be used as a single tool for diagnosis, meaning that results obtained with this method should be carefully interpreted [25, 26]. This is because the measurements of the oocysts may undergo variations due to changes in metabolism of the parasites or birds and even in the value of the shape morphometric indices of the oocysts that may overlap leading to misleading conclusions regarding the species [27]. Despite the limitations of morphometric techniques, there are some reports that indicated that oocyst morphometry could also be a sensitive method for the discrimination of* Eimeria* species of poultry in field trials as it shows high degree of agreement with the molecular methods [28–31]. The use of molecular techniques is the most specific and rapid way of diagnosing* Eimeria* infection especially when it involves several species occurring concurrently [32–34]. However, in developing countries like Nigeria very few laboratories have the facilities and personnel to carry out these molecular techniques for routine diagnosis of coccidiosis. The study also showed that the gross lesions associated with* Eimeria* infection in Japanese quails were limited to mainly ballooning of the caeca. The only clinical signs observed were diarrhea. The reports of previous works seem to be at variance with the present observation as regards the pathogenicity of* Eimeria* in quails. Mazurkiewicz et al. [35] reported a wide range of clinical signs such as lack of appetite, ruffled feathers, uncoordinated movements, inhibition of laying, and loss of weight in naturally infected young and mature quails reared at the laboratory. In young Japanese quailsinfected with* Eimeria bateri*, mild loss of weight, anorexia, and softening of feces were observed and were considered mild and easy to overcome [36]. Tsunoda and Muraki [37] also reported low pathogenicity in Japanese quails experimentally infected with 1 × 10^5^ oocysts of* Eimeria uzura* with signs limited to diarrhea and anemia with no mortality was reported. However, Ruff et al. [38] used pure and mixed cultures of* E. uzura *to infect quails and reported mortality, lower weight gain, and poor reproductive performance. Several factors such as environment, differences in the parasite strain, and management system may be responsible for the discrepancies in the observation of the various works. The findings from this study show that merozoites are the main endogenous stages of the parasites found in the small intestine and schizonts in the large intestine. The absence of other pathogenic stages such as the gametocytes means that the pathology of the infected quails will be mild. Histopathological lesions were located in the villi. These observations resemble those of Norton and Peirce [36], Mazurkiewicz et al. [35], and Tsutsumi [9] as regards the site of infection. Desquamation of epithelial lining and caecal necrosis were also observed in the study.

In conclusion, four* Eimeria *species were identified in the study, only* Eimeria bateri* speciated as it conformed to the guideline used. Due to the limitations of speciation using the oocysts morphometry technique, there is the need for further studies to be carried out using molecular techniques to properly identify the unknown species of* Eimeria* which were detected in the study. The study also showed that the gross and histopathological changes in intestinal tract pointed to the serious effect of* Eimeria *species in quails. This observation has an important value since there is a paucity of information on the pathogenicity of* Eimeria* spp. in quails.

## Figures and Tables

**Table 1:** Morphological characteristics and speciation of *Eimeria* in Japanese quail.

Species	Oocyst size (*μ*)			Shape index	Morphology	Polar granule	Oocyst wall	Confirmed species
	Length (Mean ± StE.)	Width (Mean ± StE.)	Range					
(a) *Eimeria *spp.	22.20 ± 0.58	16.38 ± 0.42	(18.20–25.48) (14.56–18.20)	1.36	Subspherical	Present	Double	*Eimeria bateri *
(b) *Eimeria *spp.	22.36 ± 1.67	15.08 ± 0.95	(14.56–25.48) (10.92–18.20)	1.48	Ovoid	Absent	Double	Unknown
(c) *Eimeria *spp.	16.64 ± 1.08	16.12 ± 0.74	(14.56–21.84) (14.56–18.20)	1.03	Ellipsoidal	Absent	Double	Unknown
(d) *Eimeria *spp.	20.57 ± 0.40	14.74 ± 0.56	(18.20–21.84) (10.92–21.84)	1.40	Subspherical	Absent	Double	Unknown
